# Diet, faecal pH and colorectal cancer.

**DOI:** 10.1038/bjc.1983.165

**Published:** 1983-07

**Authors:** W. van Dokkum, B. C. de Boer, A. van Faassen, N. A. Pikaar, R. J. Hermus


*Br. J. Cancer* (1983), **48**, 109–110

**Short Communication**

### Diet, faecal pH and colorectal cancer

W. van Dokkum, B.C.J. de Boer*, A. van Faassen†, N.A. Pikaar & R.J.J. Hermus

*Institute CIVO-Toxicology and Nutrition TNO, P.O. Box 360, 3700 AJ Zeist, The Netherlands.*

Thornton (1981) put forward the hypothesis that acidification of the colon by dietary fibre may prevent the degradation of bile acids or cholesterol into (co-)carcinogens and so may contribute to the prevention of colorectal cancer. This hypothesis might gain in importance if the colonic pH could be manipulated through diets without an excessive amount of high fibre foods like 5 to 7 oranges each day as in the study of Walker *et al.* (1979).

We performed a comparative study on the influences of a mixed western diet, a lactovegetarian diet and a vegan diet on mineral metabolism and colonic function. Twelve apparently healthy male Caucasian volunteers (students, age 20–25 years) were carefully selected for trustworthiness. They consumed the 3 different diets for 20 days each in randomized order. They were housed in the Institutes' controlled metabolic ward for the duration of the study, i.e. 60 days. They consumed all their meals in the Institute but continued their normal daily activities. Their diets consisted of constant and standardized meals and drinks. We measured blood parameters, mineral balances, faecal concentrations of bile acids, fatty acids, dietary fibre components and bacteria, transit time and faecal pH. Faecal pH was measured in freshly-passed stools of the 13th and 15th day of a diet. We assumed that this pH reflects that of the colonic content. Three regions of a stool were sampled to give 0.5–3 g faeces. The pH was measured with an electrode after 2-fold and after 3-fold dilution with distilled water.

The mean pH of the 3-fold diluted faeces of the volunteers consuming the vegan diet (median value, 6.75) was significantly lower than when consuming the lactovegetarian (median value, 7.2) or the mixed western (median value, 7.35) diet ($P<0.01$ when tested with signed-ranks test; Figure 1). The mean difference between the pH of the stool on the 13th and on the 15th day was 0.25, ranging from $-0.5$ to $+0.7$. The pH of the 3-fold dilution was consistently higher than that of the 2-fold dilution: mean value of 0.1 pH unit. The pH was also measured in a 3-fold dilution of 4 day composites (Day 13–16) of faeces from 6 volunteers: values of 6.6, 7.2 and 7.2 were measured in the faeces of the volunteers consuming the vegan, lactovegetarian and mixed western diet respectively. These pHs are comparable with those of the freshly-passed faeces on Days 13 and 15.

**Figure 1** Median and individual values of mean faecal pH (n=2) of 12 healthy male volunteers (age 20–25 years) consuming 3 different diets during a period of 20 days each

*Faculty of Biology, University of Amsterdam.
†Koningin Wilhelmina Fonds (Netherlands Cancer Foundation).

Correspondence: W. van Dokkum.

Received 29 March 1983; accepted 17 April 1983.

© The Macmillan Press Ltd., 1983

The amounts of dietary fibre components consumed per day, as approximately measured with a modification of the Van Soest method in which pre-digestion with pancreatin was applied, are shown in Table I.

**Table I** Consumption of dietary fibre components from 3 different diets (g per day)

| | Cellulose | Hemicellulose | Lignin |
|---|---|---|---|
| Mixed western diet | 6.6 | 10.8 | 4.0 |
| Lactovegetarian diet | 9.2 | 12.2 | 4.6 |
| Vegan diet | 13.2 | 23.4 | 6.8 |

The relatively high intake of cellulose and of hemicellulose while on the vegan diet might be the causal factor for the low faecal pH observed. These components of dietary fibre from a mixed diet are reported as being digestible to a considerable extent. Holloway *et al.* (1978) measured a digestibility of cellulose of 80% and of hemicellulose of 96% and Ullrich *et al.* (1981) of 16 and 45% respectively. The differences probably depend on the composition of the diet.

In this connection it is interesting that Bingham *et al.* (1979) found a negative correlation between the intake of pentose from dietary fibre and the mortality of large-bowel cancer in Britain. Hemicellulose measured with the Van Soest method, contains the larger part of the pentoses, as can be concluded from the review of Kay *et al.* (1978).

We suggest that a lower faecal pH may be correlated with a lower mortality of large-bowel cancer and that faecal pH should always be considered in epidemiological studies on the role of diet in colon carcinogenesis.

### References

BINGHAM, S., WILLIAMS, D.R.R., COLE, T.J. & JAMES, W.P.T. (1979). Dietary fibre and regional large-bowel cancer mortality in Britain. *Br. J. Cancer*, **40**, 456.

HOLLOWAY, W.D., TASMAN-JONES, C. & LEE, S.P. (1978). Digestion of certain fractions of dietary fiber in humans. *Am. J. Clin. Nutr.*, **31**, 927.

KAY, R.M & STRASBERG, S.M. (1978). Origin, chemistry, physiological effects and clinical importance of dietary fibre. *Clin. Invest. Med.*, **1**, 9.

THORNTON, J.R. (1981). High colonic pH promotes colorectal cancer. *Lancet*, **i**, 1081.

ULLRICH, I.H., LAI, H.Y., VONA, L., REID, R.L. & ALBRINK, M.J. (1981). Alterations of faecal steroid composition induced by changes in dietary fiber consumption. *Am. J. Clin. Nutr.*, **24**, 2054.

WALKER, A.R.P., WALKER, B.F. & SEGAL, I. (1979). Faecal pH value and its modification by dietary means in South African Black and White schoolchildren. *S. Afr. Med. J.*, **55**, 495.


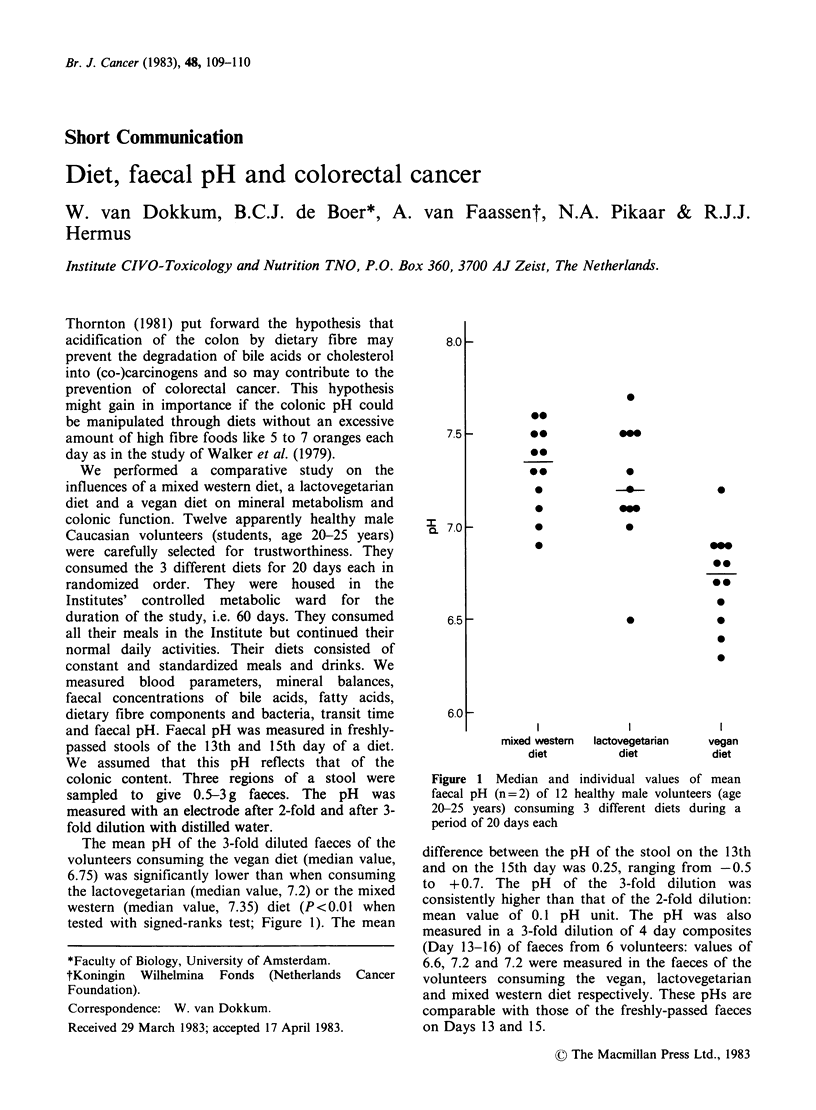

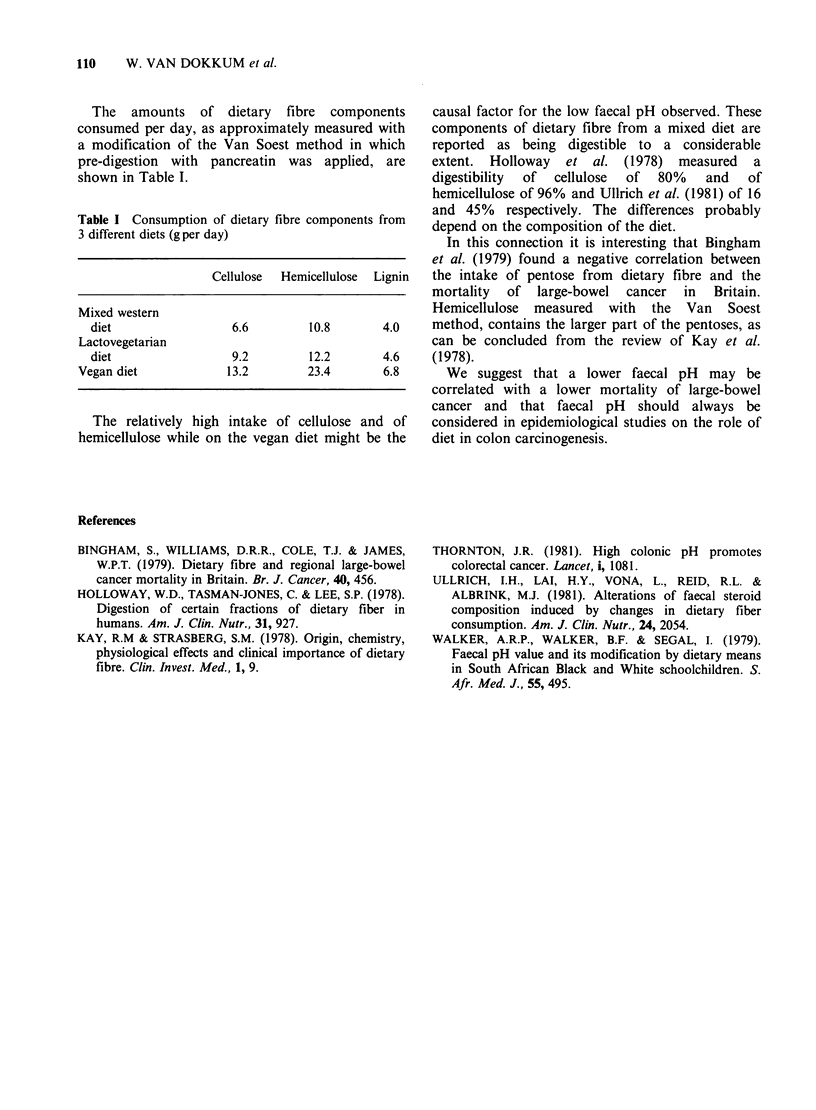


## References

[OCR_00171] Bingham S., Williams D. R., Cole T. J., James W. P. (1979). Dietary fibre and regional large-bowel cancer mortality in Britain.. Br J Cancer.

[OCR_00176] Holloway W. D., Tasman-Jones C., Lee S. P. (1978). Digestion of certain fractions of dietary fiber in humans.. Am J Clin Nutr.

[OCR_00181] Kay R. M., Strasberg S. M. (1978). Origin, chemistry, physiological effects and clinical importance of dietary fibre.. Clin Invest Med.

[OCR_00186] Thornton J. R. (1981). High colonic pH promotes colorectal cancer.. Lancet.

[OCR_00190] Ullrich I. H., Lai H. Y., Vona L., Reid R. L., Albrink M. J. (1981). Alterations of fecal steroid composition induced by changes in dietary fiber consumption.. Am J Clin Nutr.

[OCR_00196] Walker A. R., Walker B. F., Segal I. (1979). Faecal pH value and its modification by dietary means in South African black and white schoolchildren.. S Afr Med J.

